# ECDI‐fixed donor splenocytes prolong skin allograft survival by promoting M2 macrophage polarization and inducing regulatory T cells

**DOI:** 10.1096/fba.2019-00029

**Published:** 2019-10-17

**Authors:** Bo Zhou, Yuan Zhang, Dongliang Zhang, Yun Zhang, Jiangang Xie, Xuexin Zhang, Jianke Ding, Yingjun Su, Shuzhong Guo, Ran Zhuang

**Affiliations:** ^1^ Department of Immunology Fourth Military Medical University Xi'an China; ^2^ Department of Plastic and Reconstructive Surgery Xijing Hospital Fourth Military Medical University Xi'an China; ^3^ Transplant Immunology Laboratory Fourth Military Medical University Xi'an China

**Keywords:** donor‐specific tolerance, ECDI‐SP, transplantation rejection

## Abstract

Rejection is a common complication of allogeneic tissue transplantation. Fixation of splenocytes (SP) with 1‐ethyl‐3‐(3'‐dimethylaminopropyl)‐carbodiimide (ECDI) induces immune tolerance in recipients post‐transplantation; however, the mechanism underlying this effect remains unclear. Here, we determined the mechanisms of ECDI‐fixed donor SP (ECDI‐SP) in inducing tolerance in skin allograft transplantation. C57BL/6‐recipient mice that received Balb/c full‐thickness skin transplants with two infusions of donor‐derived ECDI‐SP, along with rapamycin showed superior skin allograft survival and lower inflammatory cell infiltration than mice that received rapamycin‐only treatment. In ECDI‐SP‐treated mice, the levels of anti‐inflammatory cytokines such as interleukin (IL)‐10 in sera were markedly increased, whereas the expression of inflammatory cytokines was significantly suppressed. Splenic macrophages were significantly polarized to the alternative activated macrophage (M2) phenotype, with expansion of CD4^+^Foxp3^+^ regulatory T cells (Tregs) in the spleen and draining lymph nodes. Allostimulatory activity of ECDI‐SP in vitro and donor‐specific ex vivo hyporesponsiveness were observed. C57BL/6 macrophages engulfed allogeneic Balb/c‐derived ECDI‐SP, polarized to the M2 phenotype, with pronounced cAMP response element‐binding (CREB) protein phosphorylation. By facilitating increased IL‐10 expression, ECDI‐SP induced M2 polarization and Treg production, inhibiting effector T‐cell proliferation. Thus, ECDI‐SP modulates macrophage M2 polarization by increasing CREB phosphorylation and promoting Treg production to suppress allogeneic skin graft rejection.

**Abbreviations**
APCs: antigen‐presenting cells
Arg1: arginase 1
CFSE: carboxyfluorescein diacetate succinimidyl ester
CREB: cAMP response element binding
ECDI: 1‐ethyl‐3‐(3'‐dimethylaminopropyl)‐carbodiimide
IFN‐γ: interferon gamma
IL: interleukin
iNOS: induced nitric oxide synthase
LPS: lipopolysaccharide
mAb: monoclonal antibody
MCP‐1: monocyte chemotactic protein 1
PBS: phosphate‐buffered saline
PMs: peritoneal macrophages
qPCR: quantitative PCR
SP: splenocytes
TNF: tumor necrosis factor
Tregs: regulatory T cells

**Highlights**

- Infusion ECDI‐fixed donor SP may induce immune hyporesponsiveness and prolong survival of skin allografts in mice.
- The ECDI‐SP induced anti‐inflammatory profile may play an immunosuppressive role.
- Establishment of donor‐specific hyporesponsiveness is related to ECDI‐SP infusion‐induced macrophage M2 polarization and Treg expansion.

## 1 INTRODUCTION

Allograft rejection is a major obstacle for allotransplantation worldwide and is triggered by the highly antigenic skin compartments of donor tissues, the prevention of which requires long‐term and intensive immunosuppression.^1^ At present, mainstream anti‐rejection drugs mainly act on T cells to inhibit their activation and subsequent rejection. Although these drugs alleviate anti‐graft immunity, they cause a variety of side effects, which greatly limits the development of organ transplantation. Therefore, the development of efficient anti‐immune rejection drugs or therapeutic methods for the induction of donor‐specific tolerance is highly desirable. They can promote the production of tolerance antigen‐presenting cells (APCs), such as dendritic cells and M2 macrophages, which can induce donor‐specific immune tolerance.^2^

ECDI‐fixed splenocytes (ECDI‐SP) have been used as a minimally toxic immunosuppressive method for treating autoimmune diseases in clinical studies.^3^ The delivery of donor antigen‐pulsed splenic APCs artificially fixed with ECDI is a safe and powerful approach to induce antigen‐specific tolerance.^4^, ^5^ This system has been shown to induce donor‐specific tolerance effectively in islet transplantation, thus prolonging cardiac allograft survival in mice.^6^, ^7^ Possible mechanisms underlying this effect include the induction of cell apoptosis and antigen‐specific tolerance through both direct and indirect antigen presentation following ECDI treatment.^8^ However, the mechanisms by which ECDI‐treated cells induce immune tolerance are not yet well understood.

Macrophages initiate the innate immune response and are responsible for processes including phagocytosis and antigen presentation.^9^ After the phagocytosis of antigens, macrophages are polarized to the M1 (classic macrophages) or M2 (alternative macrophages) phenotypes, which determines the direction and intensity of subsequent specific immune reactions.^10^ Many studies have shown that M1 macrophages promote inflammation, whereas M2 macrophages inhibit inflammation through the secretion of interleukin (IL)‐10 and other anti‐inflammatory cytokines.^11^, ^12^ ECDI‐fixed donor cells rapidly undergo apoptosis, and specific antigens can be processed and presented by macrophages. Furthermore, M2 macrophages contribute to non‐specific immunity and are important in inducing regulatory T cells (Tregs). Studies have also indicated that the depletion of Tregs abolishes ECDI treatment‐induced tolerance, suggesting that the presence of Tregs is crucial for inducing donor‐specific tolerance.^13^

This study focused on macrophage polarization after the cells engulfed ECDI‐SP. In vivo experiments demonstrated that ECDI‐SP treatment prolonged the survival of skin allografts. ECDI‐SP polarized macrophages to the M2 type in a mice skin graft transplantation model. Additionally, the percentage of Tregs and the level of IL‐10 in ECDI‐SP‐treated mice sera increased significantly, whereas the expression of IL‐6 and other inflammatory factors decreased. Using an in vitro co‐culture system, we found that ECDI‐SP effectively induced phosphorylation of the transcription factor cAMP response element‐binding protein (CREB) and promoted the polarization of M2 macrophages and differentiation of Tregs. The expression of IL‐10 increased significantly and that of inflammatory cytokine IL‐6 decreased significantly after the macrophages were exposed to ECDI‐SP. These findings reveal the mechanism underlying the prolongation of allograft survival following treatment with donor ECDI‐SP, providing a theoretical basis for the development of new clinical therapeutic techniques.

## 2 MATERIALS AND METHODS

### 2.1 Mice

Male Balb/c (H‐2^d^) and C57BL/6 (H‐2^b^) mice at 8‐10 weeks of age were obtained from the Laboratory Animal Center of Fourth Military Medical University (Xian, China). All mice were housed in specific pathogen‐free conditions. Protocols were approved by the Animal Experimental Committee of Fourth Military Medical University.

### 2.2 Generation of mice peritoneal macrophages and splenic mononuclear cells

Peritoneal macrophages (PMs) were isolated from the abdominal cavities using phosphate‐buffered saline (PBS; Hyclone) and cultured in 6‐well plates with 10% fetal calf serum (Gibco) and Roswell Park Memorial Institute (RPMI) 1640 culture medium (containing 90 U/mL penicillin and 90 µg/mL streptomycin) for 4 hours. Mice in the positive control groups were treated with lipopolysaccharide (LPS; Sigma), plus interferon (IFN)‐γ or IL‐4 (R&D) for M1 or M2 polarization, respectively. SP or ECDI‐SP and macrophages were co‐cultured at a ratio of 5:1. After 12 hours of co‐culturing, phagocytes were detected; after 24 hours, macrophage polarization was detected using flow cytometry and real‐time quantitative PCR (qPCR).

### 2.3 Preparation of ECDI‐SP

ECDI‐SP were prepared and used as reported previously.^5^ Briefly, the spleens from Balb/c mice were processed to obtain single‐cell suspensions. Next, mononuclear cells were isolated by density separation in mouse lymphocyte separation medium (density: 1.081 g/mL, Dakewei Biotech). Splenic mononuclear cells (1 × 10^8^) were re‐suspended in 1 mL of PBS containing 30 mg/mL ECDI (Sigma) on ice for 1 hour, inverted and gently shaken, and then washed twice with PBS (ECDI‐SP preparation). Control splenic mononuclear cells were treated with PBS only (SP preparation). Each recipient mouse was injected with a 200 µL suspension containing 1 × 10^7^ untreated SP, or treated ECDI‐SP, via the angular vein 7 days before and 1 day after skin transplantation.

### 2.4 Skin transplantation and ECDI‐SP treatment

The skin allograft and ECDI‐SP injection were performed as described previously.^4^, ^5^ Briefly, 1 × 1 cm full‐thickness skin, obtained from the tails of Balb/c mice, were grafted onto beds prepared on the lateral flanks of C57BL/6 recipient mice. Bandages and adhesive tape were used to protect the graft sites. Bandages were removed 10 days after transplantation. The skin allografts were monitored and photographed daily after the bandages were removed. Rapamycin was administered intraperitoneally at 1 mg/kg/d to recipient mice 1 day prior to and 8 days after transplantation as basal immune‐inhibition. ECDI‐SP (1 × 10^7^) were injected intravenously 7 days prior to and 1 day after skin transplantation (Rapa + ECDI‐SP group, n = 6). Control skin allograft mice were treated with rapamycin alone (Rapa group, n = 6). At the end of rejection, recipient C57BL/6 mice splenocytes were isolated and labeled with carboxyfluorescein diacetate succinimidyl ester (CFSE) to assess T‐cell proliferation. Balb/c‐derived SP were incubated with mitomycin C and used as stimulator cells.

### 2.5 Graft histology

The skin grafts, spleens, axillary lymph nodes, and inguinal lymph nodes of the mice were harvested, fixed in 10% buffered formalin, and embedded in paraffin at the time of graft rejection. The tissues were sectioned and stained with hematoxylin and eosin (H&E) to assess the morphological changes according to the standard protocols. Rejection criteria were based on those outlined in the Banff 2007 Working Classification system.

### 2.6 Luminex assay

Sera from the C57BL/6 recipients were collected at the time of rejection. Supernatants from cells used in the one‐way mixed lymphocytic reaction were also collected at day 5. The concentrations of mouse cytokines IL‐4, IL‐10, IL‐2, IL‐12p70, tumor necrosis factor (TNF)‐α, IFN‐γ, MCP‐1, IL‐6, IL‐1β, and IL‐17A were detected using the Luminex 100 assay system (R&D Systems, Cat #: LXSAMSM) according to the manufacturer's protocol.

### 2.7 Analysis of macrophage phagocytosis

Before ECDI treatment, SP were fluorescently labeled with CFSE (eBioscience). Briefly, SP were suspended in PBS at a concentration of 1 × 10^7^ cells/mL and incubated at 37°C for 30 minutes with 5 µmol/L CFSE. Next, the SP were washed twice with serum‐free RPMI 1640 medium, resuspended at 5 × 10^6^ cells/mL in 10% fetal calf serum culture medium, and used for ECDI treatment as described previously.^5^ Macrophages were co‐cultured with ECDI‐SP or SP for 12 hours. Cells were blocked with anti‐CD16/32 antibody for 15 minutes, and then stained with an APC‐conjugated monoclonal antibody (mAb) against F4/80 (eBioscience) for 30 minutes. The cells were then analyzed using a BD Accuri C6 Flow Cytometer (BD Biosciences) and analyzed using FlowJo software. The F4/80 and CFSE double‐positive cells were considered as macrophages that had ingested SP or ECDI‐SP.

### 2.8 Flow cytometric analysis

For macrophage polarization analysis, macrophages were primed by co‐culturing with SP or ECDI‐SP for 24 hours, respectively. The immune cellular staining and flow cytometry analysis were performed as reported previously.^14^ Briefly, the primed macrophages were stained with APC‐conjugated anti‐F4/80 mAb, Percp‐Cy5.5‐conjugated anti‐CD206 mAb, and fluorescein isothiocyanate‐conjugated anti‐CD11c mAb (Biolegend) to assess the macrophage polarization. For the staining of Tregs, the cells were stained with Percp/Cy5.5‐CD4, fixed, and permeabilized in fixation/permeabilization buffer (eBioscience), and then stained with Alexa Fluor 488‐Foxp3 antibody (eBioscience). All data were collected on the BD Accuri C6 Flow Cytometer.

### 2.9 Real‐time quantitative pcr analysis

Total mRNA was extracted with TRIzol reagent (Invitrogen) and reverse‐transcribed to cDNA using cDNA synthesis kit (Fermentas, Inc). The primers used for this study were supplied by Tsingke, Inc (Xi'an, China) and are shown in the (Table S1). Following normalization to the *Gapdh* gene, the levels of target gene expression were calculated using the comparative threshold cycle method.

### 2.10 Western blotting

RAW264.7 macrophages were co‐cultured with SP or ECDI‐SP for 24 hours. Untreated cells were used as the blank control. Protein was extracted for SDS‐PAGE on a tris‐glycine gel and transferred to polyvinylidene fluoride membranes. Membranes were incubated with primary antibodies against β‐actin, CREB, or phos‐Ser133 CREB (Cell Signaling Technology) overnight at 4°C, followed by incubation with horseradish peroxidase‐labeled secondary antibodies for 1 hour at room temperature. Protein bands were visualized using enhanced chemiluminescence substrate (Pierce).

### 2.11 One‐way mixed lymphocytic reaction

Splenic mononuclear cells from C57BL/6 mice were labeled with CFSE, as described previously, to assess T‐cell proliferation. Balb/c‐derived SP were incubated with mitomycin C and used as stimulator cells. In System 1, 10^5^ C57BL/6 SP were co‐cultured with 2 × 10^5^ stimulator cells (responder cells: stimulator cells = 1:2) in a round‐bottom 96‐well plate. At the same time, Balb/c‐derived ECDI‐SP were added to the system in different ratios (three groups, ECDI‐SP: responder cells = 0.5:1, 1:1, and 2:1, respectively). In System 2, C57BL/6 macrophages were firstly incubated with ECDI‐SP for 12 hours, followed by co‐culturing with responder and stimulator cells (three groups, macrophages: responder cells = 0.5:1, 1:1, and 2:1, respectively). After co‐culturing for 5 days, the cells were harvested, and T‐cell proliferation was measured by flow cytometry with CFSE dilution.

For the CFSE staining and proliferation assays, lymphocytes were stained with the fluorescent dye CFSE (2 μmol/L) at 37°C for 15 minutes. The reaction was stopped by adding complete RPMI1640 medium supplemented with 15% fetal bovine serum for 5 minutes. CFSE binds to cellular proteins. When the lymphocytes divide, they share the dye equally between daughter cells so each daughter cell will have half the amount of total fluorescence of the parent cells. After 4 days’ co‐culture, the percentage of proliferating lymphocytes was analyzed by flow cytometry. The number of divisions was measured and quantitatively indicate the proliferation level.^14^ T cells were collected and stained with PE‐conjugated‐anti CD4 mAb (eBioscience) for 30 minutes. The cells were analyzed using a BD Accuri C6 Flow Cytometer (BD Biosciences) and the data were analyzed using the FlowJo software. CD4 + cells were selected, and the proliferation was analyzed by detection of CFSE proliferative peaks.

### 2.12 Statistical analysis

Graft survival was calculated using the Kaplan‐Meier method, and the results were compared between the groups using the log‐rank test. Student's *t* test and ANOVA were used for continuous variables. Data are presented as the mean ± SD, where results with *P* < .05 were considered statistically significant. Statistical analyses were performed using SPSS 16.0 software (SPSS, Inc).

## 3 RESULTS

### 3.1 ECDI‐SP infusions efficiently prolong allograft skin survival

To investigate the effects of ECDI‐SP in vivo, an allogeneic mouse skin graft model was established. Balb/c donor‐derived ECDI‐SP were injected into C57BL/6 recipient mice on days −7 and +1 via the angular vein. Rapamycin was administered daily from days −1 to +8 intraperitoneally as the basic immunosuppress treatment.

**Figure 1:**
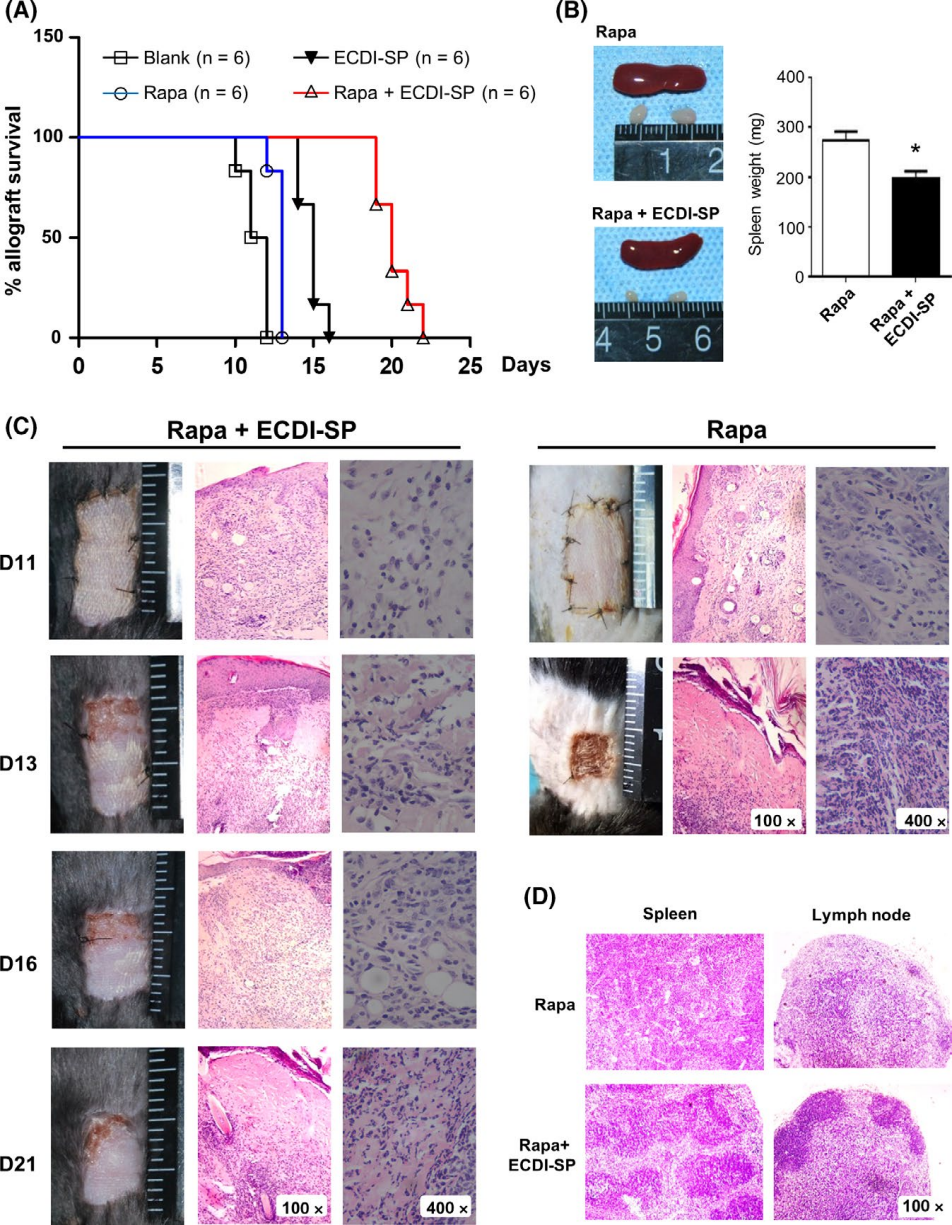
Combination treatment of ECDI‐splenocytes (SP) plus rapamycin efficiently prolonged skin allograft survival and reduced inflammation infiltration in vivo. Balb/c and C57BL/6 mice were chosen as donors and recipients, respectively. Balb/c full‐thickness skin was grafted onto the lateral flanks of C57BL/6 mice. Rapamycin (Rapa group, n = 6), ECDI‐SP (ECDI‐SP group, n = 6) and rapamycin plus ECDI‐SP (Rapa + ECDI‐SP group, n = 6) were injected via the angular vein on day −7 and +1 post‐transplantation, respectively. The mice treated with rapamycin plus ECDI‐SP had a longer allograft survival time than mice treated with rapamycin alone. (A), The Kaplan‐Meier survival curve of skin allografts from the four groups is presented as days post transplantation and relative survival. (B), Photos were taken at different time points post‐transplantation. For the biopsy, the allograft skin was removed and stained with hematoxylin and eosin (H&E) at different times post‐transplantation. (C), Recipient mice were euthanized on the day that the allograft skin was rejected, then dissected for spleen weight. (D), H&E staining of the spleen and lymph nodes of the skin graft. Upper panels: dense lymphocytic infiltrates with the destruction of the structure in the control group. Lower panels: the protective effect of ECDI‐SP infusion. Data are representative of two independent experiments with similar results. Rapa + ECDI‐SP treatment versus Rapa treatment only, *P < .05

Mice treated with ECDI‐SP plus rapamycin had superior rates of allograft survival compared to mice treated with rapamycin only (Rapa group). The skin allografts in the Rapa group were rejected on day 13 post‐transplantation, whereas the allografts in the Rapa + ECDI‐SP group showed no or minor rejection on day 13. Kaplan‐Meier survival analysis demonstrated statistically significant differences in survival time between the mice of the two groups. ECDI‐SP plus rapamycin therapy prolonged graft survival (median survival time = 20 days), whereas the median survival with rapamycin treatment alone was only 13 days (Figure 1A). Signs of rejection in the skin allografts in the Rapa + ECDI‐SP group were observed about 21 days post‐transplantation (Figure 1B). All allografts in the Rapa + ECDI‐SP group were rejected after 24 days post‐transplantation. H&E staining showed results that were similar to those made via the observation of skin allografts. The disintegration of skin structure, epidermis necrosis, and inflammatory cell infiltration were observed in the skin allografts of the control group 13 days post‐transplantation, whereas the skin allografts from the Rapa + ECDI‐SP groups had structural integrity and decreased inflammation infiltration. Compared to the rapamycin treatment alone, Rapa + ECDI‐SP had a clear effect on the inflammation of the spleen and lymph nodes, with reduced spleen and draining lymph node sizes upon gross inspection (Figure 1C). H&E staining showed that mice in the Rapa + ECDI‐SP group also exhibited mostly slight lymphadenovarix (Figure 1D). These data indicate that ECDI‐SP inhibited immunological rejection and inflammation infiltration, prolonging the survival of skin grafts in recipient mice.

### 3.2 Ecdi‐sp reduces inflammation

**Figure 2:**
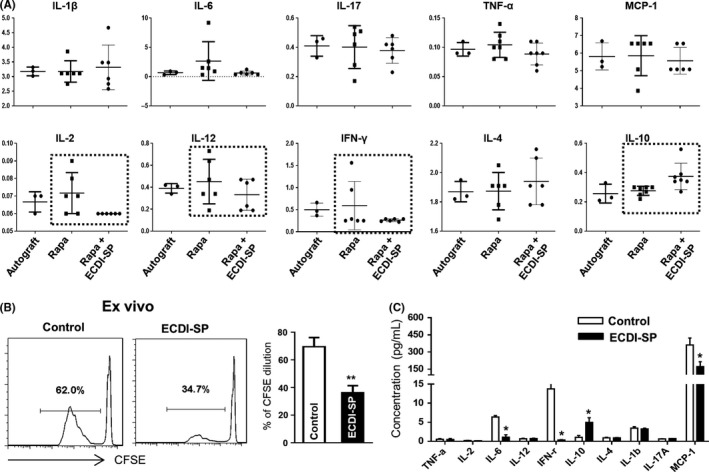
ECDI‐splenocyte (SP) treatment efficiently reduced inflammation in mice. After skin allograft transplantation, inflammation‐associated cytokines were detected to evaluate the effect of ECDI‐splenocyte treatment. (A), Sera were collected from recipient mice at day 13 post‐transplantation for Luminex cytokines analysis. The results indicated that IL‐2, IL‐6, MCP‐1, and IFN‐γ levels decreased significantly, whereas the IL‐10 levels increased significantly with the ECDI‐SP plus rapamycin combination treatment (n = 6 for each group). Data were pooled from two independent experiments, *P < .05 versus rapamycin treatment only. (B), Splenic mononuclear cells from recipients were collected at the end of skin allograft rejection to set up one‐way mixed lymphocytic reactions for 4 days. T‐cell proliferation was then measured by flow cytometry with CFSE dilution and the supernatants of the mixed lymphocytic reaction were collected for the Luminex assay. After treatment with ECDI‐SP plus rapamycin, ex vivo recipient T cells showed lower rates of proliferation than mice treated with rapamycin alone (control group). (C), ECDI‐SP therapy decreased the pro‐inflammation cytokines IL‐6, MCP‐1, and IFN‐γ, and upregulated the levels of tolerance‐inducing cytokine IL‐10 compared to that of the rapamycin‐only control group. Data in B and C are representative of three independent experiments, *P < .05, **P < .01, versus the control group

To study the possible hyporesponsiveness mechanisms, sera were collected from mice at day 13 after skin graft rejection and the cytokine levels were determined via a Luminex assay. As shown in Figure 2A, the levels of the inflammatory cytokines IL‐6, IFN‐γ, and IL‐12 were decreased, whereas the levels of the anti‐inflammatory cytokine IL‐10 increased significantly in the Rapa + ECDI‐SP group. At the end of the rejection process, the recipient C57BL/6 mice splenocytes were isolated and co‐cultured with Balb/c‐derived SP stimulator for 4 days to set up a one‐way mixed lymphocytic reaction. In this ex vivo experiment, recipient T‐cell proliferation was measured using the CFSE dilution method. As shown in Figure 2B, T‐cell proliferation was inhibited in the ECDI‐SP group. The culture supernatants were also harvested for Luminex cytokine analysis. The results also showed downregulated levels of IL‐6, IFN‐γ, and MCP‐1, as well as upregulated levels of the tolerance‐inducing cytokine IL‐10 compared to the culture supernatants of splenocytes stimulated without ECDI treatment (Figure 2C).

### 3.3 ECDI‐SP induces macrophage polarization to an M2 phenotype and increases the percentage of tregs in vivo

We examined the ability of ECDI‐SP to induce macrophage M2 polarization and increase the number of Tregs in recipient mice in vivo. On day 11 post‐transplantation, the splenocytes were harvested and stained with fluorescein‐labeled antibodies, followed by flow cytometry analysis. F4/80^+^ cells were gated as macrophages, whereas CD11c and CD206 expression patterns served as markers of macrophage M1/M2 polarization.

**Figure 3:**
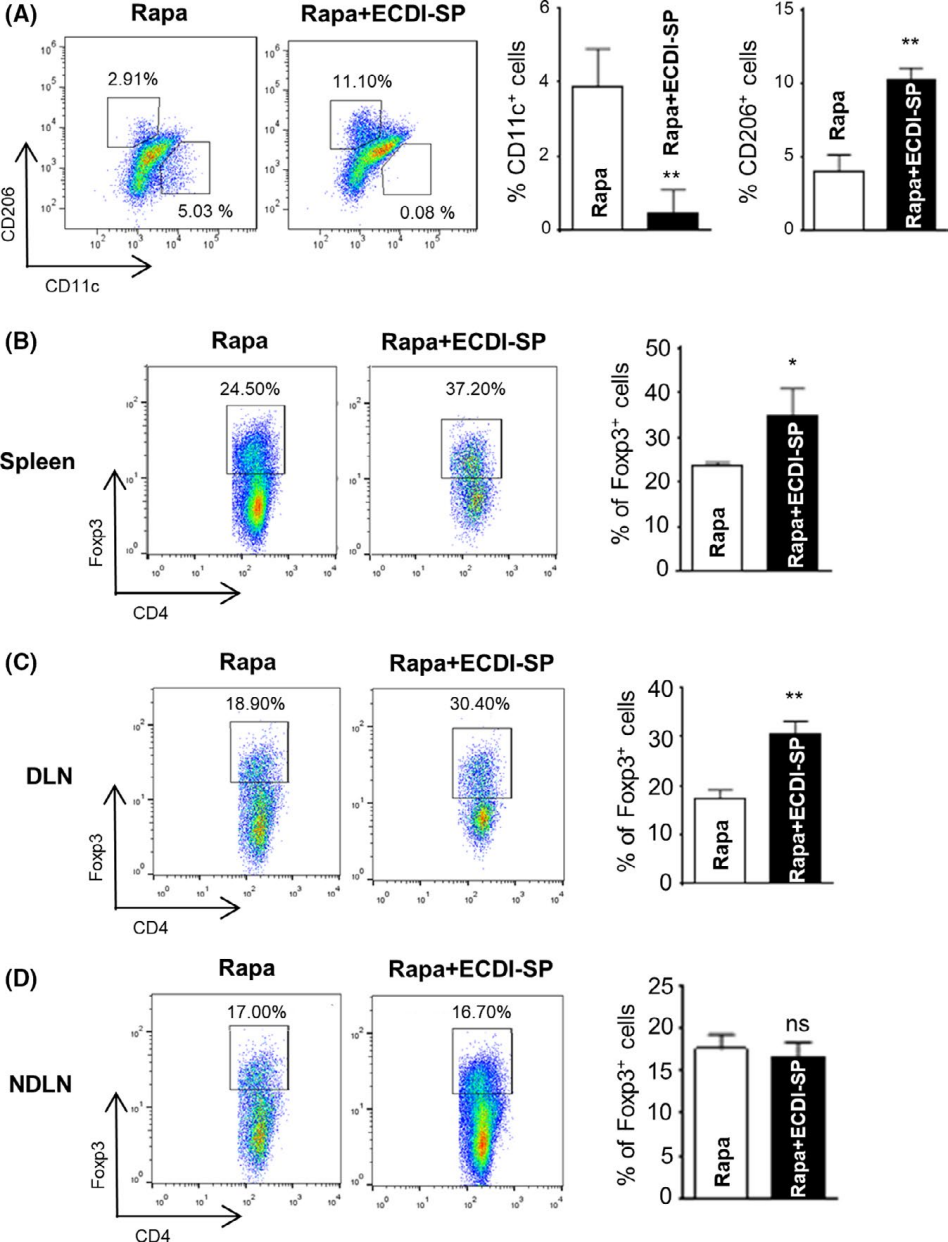
ECDI‐splenocytes (SP) induced macrophage polarization to an M2 phenotype and increased regulatory T cells (Tregs) in vivo. Spleens were extracted from C57BL/6 recipient mice on day 13 post‐transplantation. (A), Flow cytometry analysis of spleen cells showed that ECDI‐SP increased the population of M2 macrophages in vivo. (B and C), Compared to the rapamycin treatment alone, the combination of ECDI‐SP plus rapamycin significantly promoted the production of CD4^+^Foxp3^+^ Tregs both in the spleen and draining lymph nodes (DLN). (D), In the non‐draining lymph nodes (NDLN), the percentage of Tregs was not significantly different between the mice in these two groups. *P < .05, **P < .01

An increased proportion of M2 macrophages was observed in the Rapa + ECDI‐SP group, and the Rapa group had lower rates of M2 polarization (10.27 ± 0.43% vs 3.93 ± 0.68%, Figure 3A; Figure S1A). On the other hand, Rapa + ECDI‐SP treatment was found to reduce the percentage of CD11c^+^ M1 cells. In addition, compared to the mice in the Rapa group, Rapa + ECDI‐SP therapy significantly increased the population of CD4^+^Foxp3^+^ Tregs both in the spleen (Figure 3B; Figure S1B) and draining lymph nodes (Figure 3C; Figure S1C) of recipient mice. The percentages of Tregs in non‐draining lymph nodes did not show any significant differences between the Rapa + ECDI‐SP and Rapa groups (Figure 3D).

### 3.4 Macrophages engulfing ECDI‐fixed splenocytes show M2 polarization

**Figure 4:**
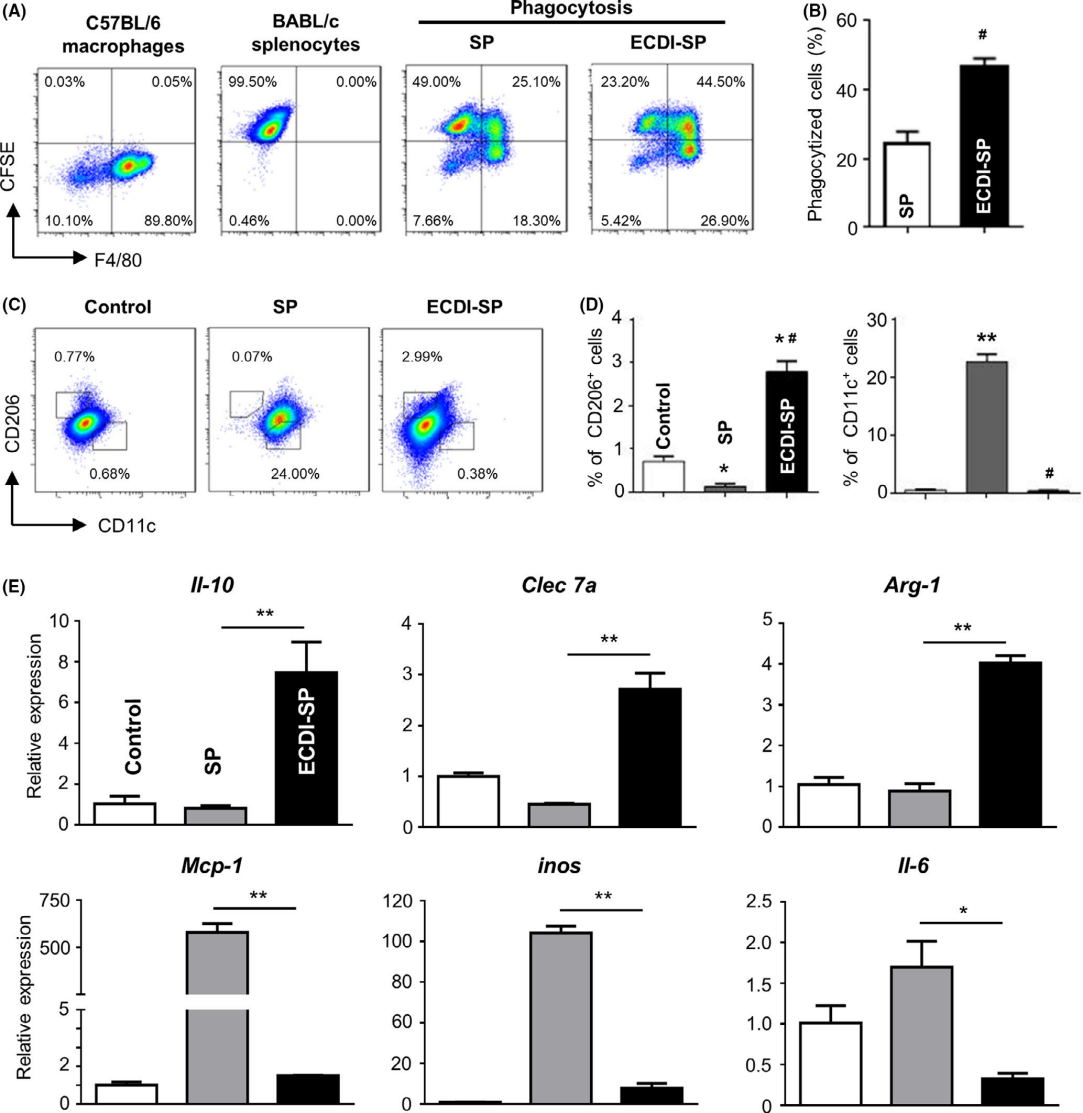
Macrophages phagocytized ECDI‐fixed donor cells and polarized cells to an M2 phenotype in allograft recipients. Balb/c splenocytes (SP) or ECDI‐SP were fluorescently labeled with carboxyfluorescein diacetate succinimidyl ester (CFSE) and co‐cultured with C57 peritoneal macrophages for 12 h. The macrophages were analyzed by F4/80 staining and flow cytometry, and total mRNA was extracted for qPCR analysis. (A), Flow cytometry results for CFSE and F4/80 double‐positive cells, indicating phagocytosis of ECDI‐SP or SP by allogeneic macrophages. (B), Percentage of macrophages with phagocytized cells in recipient mice. (C), Peritoneal macrophages remained at rest (control), co‐cultured with SP or ECDI‐SP for 12 h, then the polarization phenotypes of peritoneal macrophages were determined by CD11c/CD206 staining and flow cytometry analysis. (D), The percentages of CD11c^+^ or CD206^+^ cells in F4/80^+^ macrophages were calculated. Data are representative of three independent experiments. *P < .05, **P < .01, ^#^ *P* < .01 versus the SP group. (E), Polarization biomarker mRNA levels of *Arg1, IL‐10, Clec7a, IL‐6, inos*, and *Mcp‐1* were determined by qPCR. *P < .05, **P < .01, versus the SP group

Balb/c‐derived SP or ECDI‐SP (H‐2^d^) were labeled with CFSE and then co‐cultured with C57BL/6 peritoneal macrophages (H‐2^b^) at a ratio of 5:1 for 12 hours. Macrophages were collected and surface‐stained for F4/80. Determining the percentage of CFSE and F4/80 double‐positive cells revealed that C57BL/6 macrophages had the ability to phagocytose CFSE‐labeled splenocyte. As shown in Figure 4. A and B, the percentage of CFSE and F4/80 double‐positive cells was significantly higher in the ECDI‐SP group than in the untreated SP group (46.7 ± 2.4% vs 24.3 ± 3.5%, *P* < .01), which indicated that ECDI‐fixed SP were more easily phagocytosed.

Next, we assessed the macrophage polarization that phagocytosed SP or ECDI‐SP. After co‐culturing for 24 hours, C57BL/6 peritoneal macrophages were stained with antibodies against F4/80, CD11c, CD206, or isotype controls. After gating with the F4/80 antigen, macrophage polarization was determined using M1/M2 phenotype markers CD11c and CD206. Compared to the untreated control group, the macrophages treated with SP showed a significant increase in CD11c, whereas those from the ECDI‐SP group showed a significant increase of CD206 (Figure 4C,D). These results indicate that phagocytosis of ECDI‐SP promoted macrophage polarization to the M2 phenotype, whereas the SP‐treated macrophages polarized to the M1 phenotype.

The levels of macrophage polarization biomarkers were also determined by qPCR. After induction with ECDI‐SP, the expression of *Il‐10*, *Arg1*, and *Clec7a* (encodes dectin) mRNA increased significantly, whereas that of *Il‐6*, *inos,* and *Mcp‐1* mRNA significantly decreased, compared to the respective expression levels in cells induced with SP (Figure 4E). These results suggest that the phagocytosis of ECDI‐SP inhibited the production of inflammatory factors and M1 polarization, while promoting anti‐inflammatory cytokine production and M2 polarization.

**Figure 5:**
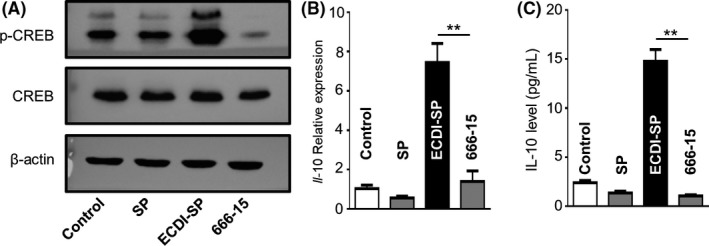
CREB phosphorylation involved in ECDI‐SP treatment. Peritoneal macrophages remained at rest (control), co‐cultured with SP or ECDI‐SP for 12 h, or with the addition of the CREB inhibitor, 666‐15. The phosphorylation and expression of CREB was determined by Western blot (A) and IL‐10 expression was detected by qPCR (B) and ELISA (C)

Furthermore, the transcription factor CREB is a critical regulator of M2 macrophage polarization. Therefore, we examined the activation of CREB in ECDI‐SP‐treated macrophages. The macrophage cell line RAW264.7, derived from Balb/c mice (H‐2^d^), showed similar phagocytosing following incubation with C57BL/6 (H‐2^b^)‐derived SP or ECDI‐SP (data not shown). As shown in Figure 5A, the phosphorylation of CREB increased significantly in ECDI‐SP‐treated macrophages compared to that after SP stimulation. Using inhibitor 666‐15 to block the CREB pathway in macrophages,^15^ the phosphorylation of CREB and the increased IL‐10 induced by ECDI‐SP were significantly suppressed (Figure 5). These results indicate that CREB may be involved in ECDI‐SP‐induced macrophage M2 polarization.

### 3.5 ECDI‐SP‐induced M2 macrophages increase treg production

**Figure 6:**
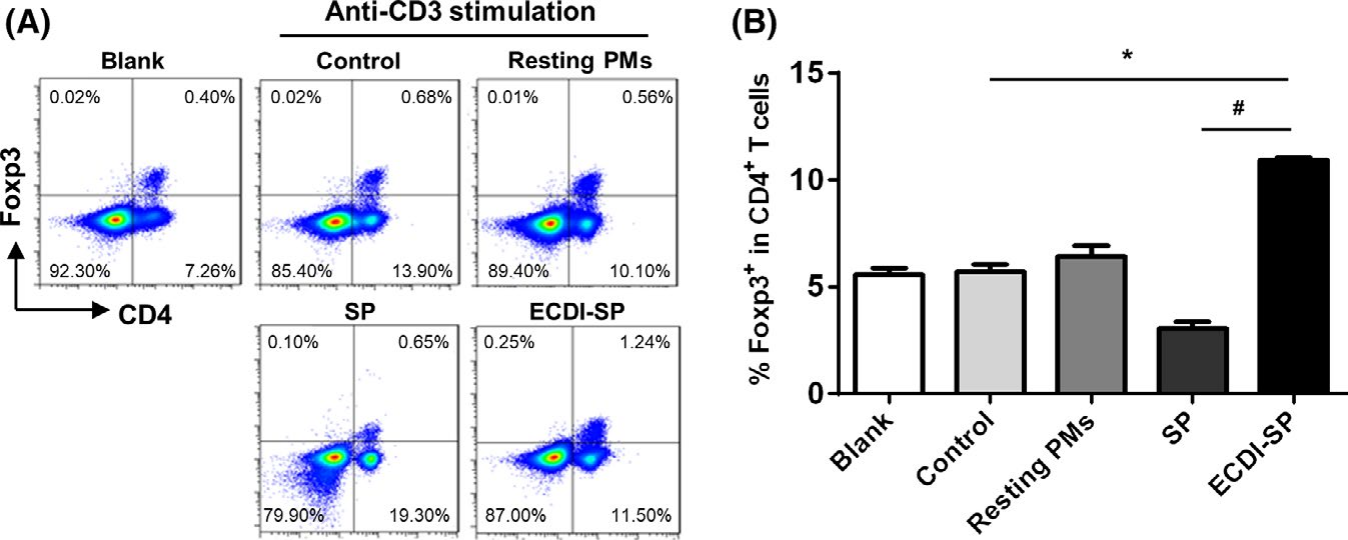
ECDI‐splenocyte‐induced M2 macrophages increased regulatory T cell (Treg) percentage. C57BL/6 macrophages were pre‐treated with Balb/c ECDI‐splenocytes (SP) to facilitate polarization. The polarized macrophages were then co‐cultured with C57BL/6 SP plus anti‐CD3 antibody stimulation for 24 h. The percentage of Treg in the C57BL/6 SP was detected and used as the blank group. Anti‐CD3 antibody treatment alone was used as the control group. (A), C57BL/6‐derived resting macrophages did not affect Treg proliferation compared to the control group. SP‐treated macrophages significantly decreased the Treg percentage. (B), Stimulation of ECDI‐SP pre‐loaded macrophages significantly increased the percentage of Tregs in CD4^+^ T cells compared to the control group. Data are representative of three independent experiments. *P < .05, versus the control group; ^#^ *P* < .01, versus the SP group

Our previous studies showed that after ECDI‐SP injection, the percentage of Tregs in allograft recipient mice increased significantly.^14^ However, the mechanism underlying this effect was unclear. In this study, the relationship between ECDI‐SP‐induced M2‐polarized macrophages and Tregs was investigated. Balb/c‐derived ECDI‐SP were incubated with macrophages from C57BL/6 mice for 24 hours to induce M2 polarization. Polarized macrophages were then used to prime C57BL/6‐derived splenocytes, followed with anti‐CD3 antibody stimulation for another 24 hours. The percentage of Tregs was determined by immunofluorescence staining and flow cytometry analysis. The percentage of Tregs in normal untreated C57BL/6 splenocytes was detected and used as the blank group. Anti‐CD3 antibody treatment only was used as the control group. In this in vitro induction system, resting macrophages did not affect Treg‐cell production compared to the control group. SP pre‐treated macrophages showed a significantly decreased Treg percentage. In contrast, the stimulation of ECDI‐SP pre‐loaded macrophages significantly increased the percentage of Tregs in CD4^+^ T cells compared to that of the control group (10.5 ± 0.3% vs 5.7 ± 0.2%, Figure 6). These results suggest that ECDI‐SP‐induced M2 macrophages promote the production of Tregs.

### 3.6 ECDI‐fixed donor cells inhibit responder T‐cell proliferation in mixed lymphocytic reaction

**Figure 7:**
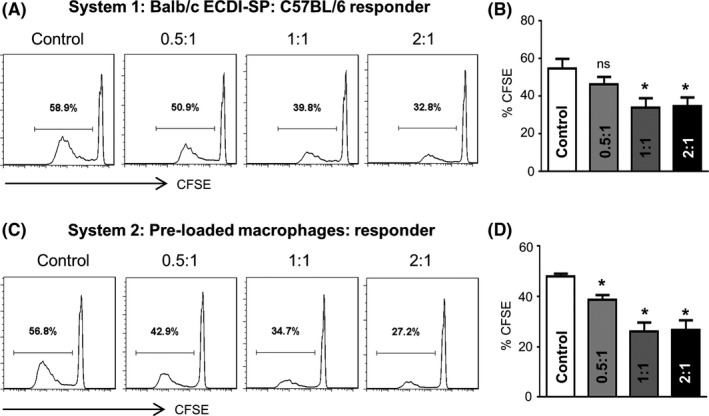
ECDI‐fixed donor cells reduced T‐cell proliferation in recipients. In the mixed lymphocytic reaction system, mitomycin‐treated Balb/c‐derived splenocytes (SP) were used as stimulator cells. C57BL/6 SP labeled with carboxyfluorescein diacetate succinimidyl ester (CFSE) were used as the responder cells. (A, B), In System 1, ECDI‐SP were added to the system in different ratios to responder T cells, as shown. ECDI‐SP inhibited responder proliferation in a dose‐dependent manner. (C, D), In System 2, ECDI‐SP were first co‐cultured and phagocytosed with C57BL/6 macrophages. ECDI‐SP pre‐loaded macrophages showed an inhibition effect in a dose‐dependent manner. Data are representative of three independent experiments. *P < .05, versus the control group

**Figure 8:**
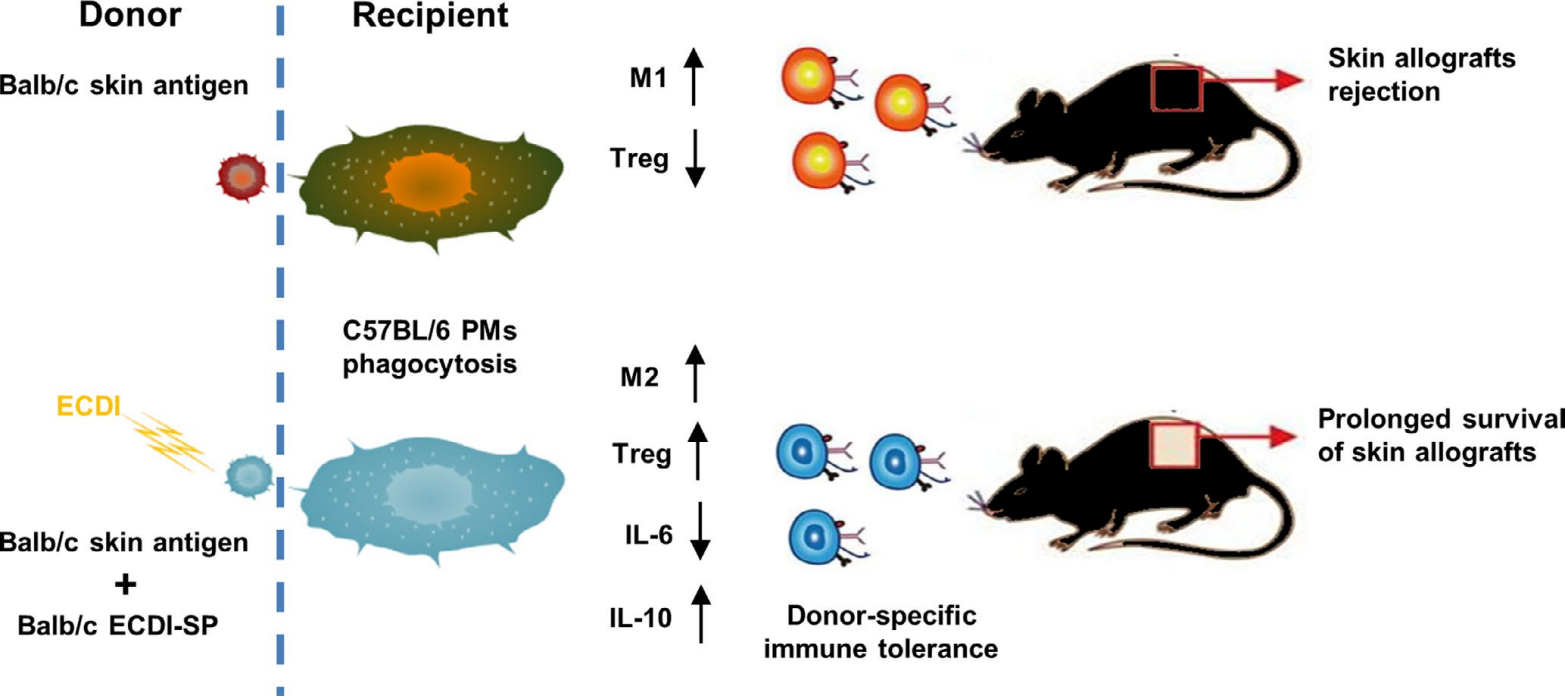
Schematic of the effect of ECDI‐splenocytes (SP) in the mouse skin allograft model. ECDI‐SP induced M2 macrophage polarization and increased the percentage of regulatory T cells (Tregs) to prolong allograft survival

In an in vitro mixed lymphocytic reaction system, Balb/c‐derived SP were pre‐treated with mitomycin and used as stimulator cells. To assess T‐cell proliferation, C57BL/6 SP were labeled with CFSE as the responder cells. In System 1, ECDI‐SP were directly added to the mixed lymphocytic reaction system in different ratios to responder T cells. Compared to the control group, ECDI‐SP significantly inhibited responder T‐cell proliferation (Figure 7A,C). In System 2, C57BL/6 macrophages were first co‐cultured with ECDI‐SP for 6 hours. ECDI‐SP pre‐loaded macrophages were then added to the mixed lymphocytic reaction system. Similar to the results of System 1, ECDI‐SP pre‐loaded macrophages inhibited T‐cell proliferation in mixed lymphocytic reaction (Figure 7C,D). These results suggest that the macrophages that had engulfed ECDI‐SP present specific tolerance antigens and may play a key role in immunosuppression. The mechanisms underlying ECDI‐SP‐induced prolongation of skin allograft survival via M2 macrophage polarization and increased Treg‐cell differentiation in mice are summarized in Figure 8.

## 4 DISCUSSION

Allogeneic skin transplantation is a lifesaving treatment for a range of health problems, including large‐scale trauma, extensive burns, and certain post‐surgical complications.^16^ However, acute rejection seriously impedes the application of skin transplantation, and inducing immune tolerance to allogeneic skin grafts is more difficult compared to solid organ transplants. Therefore, reliable strategies are necessary in order to inhibit rejection and minimize complications in allogeneic skin transplantation.^17^

The establishment of donor antigen‐specific tolerance has been shown to enhance skin allograft survival, such as pre‐transplant donor B lymphocytes, induced immature dendritic cells, and bioengineering thymus organoids to restore thymus function.^18^, ^19^, ^20^ Previous studies have showed that ECDI‐SP treatment holds great potential in inducing graft tolerance and prolonging graft survival, including islet, heart, and kidney in mice or rats.^4^, ^21^, ^22^ We previously reported on the protective effect of ECDI‐SP infusion combined with short‐term rapamycin administration in a mouse vascularized skin transplantation model.^5^ Recently, ECDI‐SP infusion combined with anti‐inflammatory agents, such as cordycepin, was shown to provide long‐term protection to mice cardiac allografts.^3^, ^23^ The use of nanoparticles as a cell‐free method for the delivery of donor ECDI‐SP antigens in murine allogeneic islet transplantation was also recently performed.^24^ However, the exact mechanism for this promising approach still needs to be elucidated.

The modulation of the host immune response through the transfusion of ECDI‐SP is a simple and non‐cytoreductive strategy to induce donor‐specific tolerance.^25^ ECDI‐treated leukocytes are known to undergo rapid apoptosis, although the precise mechanism remains unclear. Therefore, intact ECDI‐fixed cells are transient, wherein the indirect presentation of alloantigen by host APCs is likely to be the predominant tolerance mechanism. In the relationship between ECDI‐SP and T cells, it would appear that ECDI‐SP, which are presented by APCs, regulate T cells by reducing effector T‐cell signaling and enhancing Treg induction.^26^

In this study, we show that two infusions of ECDI‐treated donor SP led to the prolonged acceptance of allogeneic skin grafts in the recipients. Furthermore, ECDI‐SP therapy significantly decreased inflammatory cell infiltration into the graft, and increased IL‐10 levels while decreasing IL‐6 levels in sera. The administration of ECDI‐SP in combination with rapamycin to recipient mice markedly increased the percentage of M2 macrophages relative to that of rapamycin‐treated mice. Additionally, even at the end of the rejection process, isolated splenocytes displayed different immune responses to donor‐derived cells between the two groups, with or without ECDI‐SP treatment. In the *ex vitro* mixed lymphocytic reaction model, the splenocytes isolated from the ECDI‐SP group showed significantly higher IL‐10 production and a lower inflammatory cytokine profile. Three broad pathways control macrophages polarization: the epigenetic and survival pathways, the tissue microenvironment, and extrinsic factors, such as the cytokines released during inflammation.^27^ Here, the immunosuppressive environment created by the anti‐inflammatory cytokines induced by ECDI‐SP favored the induction of tolerance.

Previously evidence has suggested that the detection and clearance of apoptotic cells by host phagocytes generally promotes an anti‐inflammatory response, and produces anti‐inflammatory cytokines, such as IL‐10 and TGF‐β, and, at the same time, inhibit the secretion of inflammatory cytokines.^5^ Here, the phagocytosis of macrophages was measured by the CFSE‐labeling of splenocytes and flow cytometry analysis. In this study, we found that ECDI‐fixed splenocytes were easier to engulf than unfixed cells. After phagocytosis of ECDI‐SP, the profile of macrophage cytokine production followed the typical M2 phenotype, evidenced by increased levels of *Il‐10, Arg‐1*, and *Clec 7a,* and decreased levels of *Il‐6, inos*, and *Mcp‐1*. The percentage of CD206‐positive macrophages increased after co‐culturing with ECDI‐SP, whereas that of CD11c‐positive cells decreased.

Next, we investigated the molecular mechanisms involved in macrophage polarization. We found a significant amount of phosphorylation of the transcript factor CREB in ECDI‐SP pre‐loaded macrophages. Following phosphorylation and activation, the CREB pathway was found to block M1 macrophage function partly via IL‐10. Moreover, a number of CREB target genes, notably *Socs3* and *Cebpβ*, have been demonstrated to promote M2 macrophage polarization.^28^ Therefore, ECDI‐SP may promote anti‐inflammatory M2 macrophage gene expression via the upregulation of CREB. The phosphorylation of CREB is the critical molecular mechanism after engagement of ECDI‐SP by macrophages. Thus, ECDI‐SP induced M2 polarization should also be considered in allograft transplantation and is not specific for skin allografts.

Previously studies have demonstrated that this is a good strategy for inducing alloantigen‐specific tolerance, and the depletion of Tregs has been found to abolish the tolerance induction of ECDI‐treated donor cells.^29^ Moreover, the anti‐inflammatory cytokines secreted by the M2 macrophages have a positive effect on Treg proliferation and activate alloimmune responses.^30^ In this study, we investigated the role of ECDI‐SP pre‐loaded macrophages in the induction of Treg production. Interestingly, we found that CD4^+^Foxp3^+^ Tregs were markedly increased in the co‐culture with ECDI‐SP pre‐loaded macrophages in vitro. The hyporesponsiveness of recipient splenocytes may further result from the expansion of the Treg pool. Furthermore, in the in vitro mixed lymphocytic reaction model, the ECDI‐SP, regardless of whether it was added to the mixed lymphocytic reaction system directly or pre‐loaded into macrophages, inhibited the proliferation of responder T cells. This phenotype was found to be associated with the increase in CD4^+^Foxp3^+^ Tregs, which are critical in the induction and maintenance of host immune tolerance.

To summarize, we demonstrate that ECDI‐SP is a simple but highly effective donor‐specific tolerance protocol for promoting successful allogeneic skin transplantation. The tolerance of this protocol may be mediated through macrophages engulfing ECDI‐SP and displayed the M2 polarization phenotype, further promoting the expansion of Tregs.

In conclusion, this study investigated the therapeutic potential of ECDI‐fixed donor‐derived splenocytes in a mouse skin allograft model. Our data suggest that ECDI‐SP promotes macrophage M2 polarization by facilitating CREB phosphorylation, upregulating anti‐inflammatory cytokines, and enhancing Treg expansion. Therefore, ECDI‐fixed donor‐derived cells may be a highly effective strategy for improving the outcomes of allogenic transplantation.

## CONFLICT OF INTEREST

The authors declare no conflicting financial interests.

## AUTHOR CONTRIBUTIONS

R. Zhuang and SZ Guo designed research; R. Zhuang and Y. J. Su analyzed data; B. Zhou, Y. Zhang, D. L. Zhang, Y. Zhang, J. G. Xie, X. X. Zhang and J. K. Ding performed research; R. Zhuang wrote the paper.
