# Supplementary figures and images for: ECDI‐fixed donor splenocytes prolong skin allograft survival by promoting M2 macrophage polarization and inducing regulatory T cells
Source: FASEB Bioadv. 2019 Oct 17;1(11):706–18. doi: 10.1096/fba.2019-00029 (PMC6996306; doi:10.1096/fba.2019-00029)

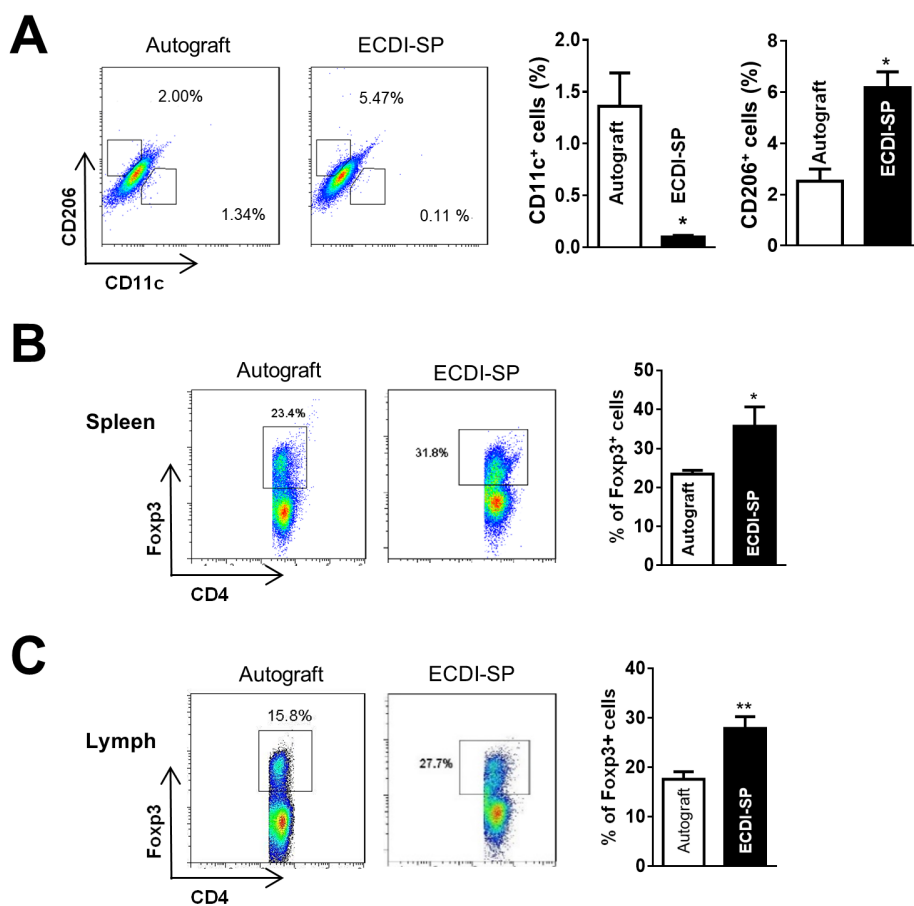

Supplement Figure S1

Supplement: Supplementary file 1 [file FBA2-1-706-s001.pdf]
