# Supplementary material for: ECDI‐fixed donor splenocytes prolong skin allograft survival by promoting M2 macrophage polarization and inducing regulatory T cells
Source: FASEB Bioadv. 2019 Oct 17;1(11):706–18. doi: 10.1096/fba.2019-00029 (PMC6996306; doi:10.1096/fba.2019-00029)
Supplement: Supplementary file 2 [file FBA2-1-706-s002.pdf]

Table S1

	sequences of primers for qPCR
Il-6 F	GAGGATACCACTCCCAACAGACC
Il-6 R	AAGTGCATCATCGTTGTTCATACA
inos F	GGCAGCCTGTGAGACCTTTG
inos R	GCATTGGAAGTGAAGCGTTTC
Clec7a F	CATCGTCTCACCGTATTAATGCAT
Clec7a R	CCCAGAACCATGGCCCTT
Mcp-1 F	AAGATGATCCCAATGAGTAGGC
Mcp-1 R	AGGTGGTTGTGGAAAAGGTAGT
Il-10 F	CAGCCGGGAAGACAATAACTG
Il-10 R	CCGCAGCTCTAGGAGCATGT
Arg1 F	TCAACACTCCCCTGACAACCA
Arg1 R	CCCAGCTTGTCTACTTCAGTC
